# Celebrating Moises A. Carreon: Special Edition Tribute to an Innovator in Membranes and Materials Science Research

**DOI:** 10.3390/membranes14110229

**Published:** 2024-11-05

**Authors:** Surendar R. Venna

**Affiliations:** The Dow Chemical Company, Freeport, TX 77541, USA; srvenna@dow.com

## 1. A Brief Overview of Professor Carreon’s Career

Professor Moises A. Carreon’s (Moisés Abraham Carreón Garcidueñas) name is highly regarded in the field of material science, especially in catalysis and membrane-based gas separation research. Professor Carreon’s story showcases both personal success and a relentless drive to push boundaries in material science. This Commemorative Special Issue in honor of Moises A. Carreon is a tribute to his legacy and the significant impact he has made on colleagues, students, and the broader scientific community. Reflecting on his achievements reminds us of the significant influence one person can have, inspiring us to continue the journey of discovery of new materials which he passionately supported.

Professor Carreon earned his bachelor’s and master’s degrees in chemical engineering from Universidad Michoacana de San Nicolás de Hidalgo (UMSNH) in Mexico. After excelling in chemical engineering as an undergraduate, he earned honors and secured a scholarship for a Ph.D. in material science at the University of Cincinnati. During his doctoral studies, Moises’ research focused on the synthesis and characterization of novel catalyst materials with unique properties for industrial application, such as selective oxidation of n-butane to maleic anhydride [1,2]. After earning his Ph.D., he pursued a career that made him a significant leader in material science. In his research career at the University of Louisville and Colorado School of Mines, Professor Carreon published more than 120 journal articles, patents, book chapters, and books. Professor Carreon was also invited to speak at several conferences and institutions.

**Figure 1:**
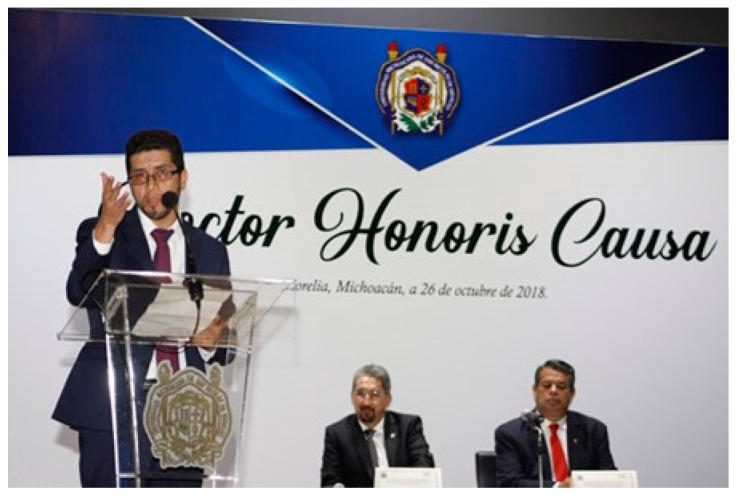
Moises A. Carreon recognized with the *Doctorate Honoris Causa* by the Universidad Michoacana (UMSNH), Mexico.

Throughout his career, Moises has received numerous awards and accolades in recognition of his contributions to material science. One of the prestigious awards received by Professor Carreon is PECASE (Presidential Early Career Award for Scientists and Engineers), the highest honor bestowed by the US government to outstanding scientists and engineers in the early stages of their independent research careers. Moises has also been awarded an honorary doctorate from Universidad Michoacana de San Nicolás de Hidalgo (Figure 1), which is the highest recognition the Mexican university confers in the fields of science, arts, and humanities, with only 71 people having received it so far, including former presidents of Mexico and Chile and Nobel chemistry laureates. Among other awards are prestigious honors from scientific societies, universities, and industry organizations, such as the National Science Foundation Career Award for his groundbreaking research in material science and engineering, the AIChE Separations Division Kunesh Award, and a list of 10 successful Mexican personalities in the USA from the magazine “Hola”. His accomplishments are a testament to his research career and his contributions to scientific community.

Carreon’s influence extends far beyond the confines of the laboratory. His work has inspired countless individuals to pursue careers in material science and engineering. In addition to his research, Moises Carreon is highly regarded for his commitment to mentoring and teaching at the University of Louisville and Colorado School of Mines. As I am the first graduate student of his academic career, I worked very closely with him in setting up the lab and working on membrane-related research activities. I admired his mentorship style. Professor Carreon provides a significant amount of freedom to come up with research ideas and execute them. At the same time, he is always available to discuss the research. He has guided numerous students and young researchers throughout his career, many of whom have gone on to make significant contributions of their own. He is a strong promotor of diversity in his research groups and throughout the engineering departments. Moises Carreon, a highly esteemed member of both the North American Membrane Society and the International Zeolite Association, has made significant contributions to scientific societies. Throughout his career, Carreon has collaborated with other leading researchers and institutions to push the boundaries of membrane technology and material science. Moises’s personal life is as inspiring as his professional and community commitments. I still recall him as a new assistant professor at the University of Louisville; he would carefully plan his day and follow it strictly, which inspired me to take up the same habits and continue to this day. He loves spending his time with his family, especially outdoors every day.

## 2. Summary of Professor Carreon’s Research

Professor Carreon’s research focuses on molecular gas separations, heterogeneous catalysis, and gas storage, aiming to tackle relevant societal issues related to energy and the environment, including carbon dioxide capture and utilization, conversion of biomass to fuels, natural gas purification, gas storage, and spent nuclear fuel treatment. He has also contributed to the fundamental understanding of the membranes materials and improved the gas separation performance by fine-tuning the material properties and surface modification of membranes. Here, we review Dr. Carreon’s research work, highlighting his contributions to the development and optimization of gas separation membranes.

Professor Carreon initiated his membrane research during his postdoctoral period at the University of Colorado, Boulder. He improved the performance of SAPO-34 zeolite membranes through novel techniques, such as using mixed structure-directing agents for CO_2_/CH_4_ separation [3]. By combining various SDAs for both seeding and membrane creation, he developed membranes that offered higher fluxes and selectivity. These membranes needed only one synthesis layer, which enhanced CO_2_ permeances and streamlined the processing. This work was further extended to reduce the cost of the membranes by replacing costly stainless-steel support with cheaper alumina support. Additionally, these membranes showed enhanced separation performance, reducing the membrane area needed for natural gas sweetening applications. High-flux SAPO-34 membranes on porous, tubular stainless-steel supports with CO_2_/CH_4_ separation selectivities greater than 200 were scaled up from 5 cm in length to 25 cm (a factor of 7.5 increase in membrane area) [4]. The preparation of the 25 cm membranes required a more dilute synthesis gel and shorter crystallization times to avoid excess build-up of zeolite crystals at the bottoms of the membrane tubes. These changes yielded 25 cm membranes with similar permeance and selectivity to the 5 cm membranes.

Professor Carreon continued his research on zeolite membranes at the University of Louisville as a faculty member. The SAPO-34 membrane performance was further enhanced by synthesizing the smaller SAPO-34 particle using crystal growth inhibitors [5] and microwave-assisted synthesis [6], which resulted in thinner membranes with enhanced CO_2_/CH_4_ separation performance. SAPO-34 seeds and membranes were functionalized with several organic amino cations to increase CO_2_ selectivity [7]. CO_2_/CH_4_ selectivity as high as 245, with CO_2_ permeances of ~5 × 10^−7^ mol m^−2^ s^−1^ Pa^−1^ at 295 K and 138 kPa, were observed for an optimum ethylenediamine-functionalized membrane, which corresponded to a ~40% increase in the separation index as compared to the nonfunctionalized SAPO-34 membrane. This membrane also showed good potential for coal power plant flue gas CO_2_ capture with improved CO_2_/N_2_ separation performance. Professor Carreon extend his zeolite membrane research to other zeolites, such as AlPO-18 zeolite membranes, for natural gas sweetening applications [8].

Natural gas reservoirs also contain trace amounts of helium and are one of the commercial sources of helium supply. Carreon’s group demonstrated that SAPO-34 membranes can effectively separate equimolar helium/methane mixtures, which was favored through molecular sieving and diffusivity differences [9]. These SAPO-34 membranes surpassed the Robeson upper bound, making these membranes attractive for helium recovery from natural gas. Professor Carreon has also developed mixed-matrix membranes (MMMs) incorporating zeolite particles, which have shown enhanced gas separation performance compared to traditional polymer membranes. Three component mixed matrix membranes composed of a polymerized room-temperature ionic liquid (poly(RTIL)), a room-temperature ionic liquid (RTIL), and SAPO-34 were synthesized and evaluated for natural gas sweetening [10]. A CO_2_/CH_4_ selectivity of 43 with a CO_2_ permeability of 202 Barrers was achieved with 30 wt% of SAPO-34.

In addition to SAPO-34 and AlPO-18 zeolite membranes, Dr. Carreon has extensively researched Zeolitic Imidazolate Frameworks (ZIFs), a subclass of MOFs characterized by their robust and thermally stable structures. Dr. Carreon’s work has highlighted the potential of ZIF-8 membranes for separating CO_2_ from natural gas streams. Research on the structural evolution of zeolitic imidazolate framework-8 (ZIF-8) as a function of time at room temperature opened up novel methods to fine-tune the gas separation properties of these materials while controlling the thickness of these membranes [11,12]. This research identified the different stages of ZIF-8 formation (nucleation, crystallization, growth, and stationary periods) and elucidated its kinetics of transformation with controlled crystallites, including nanoneedles. Utilizing this fundamental understanding of material synthesis, thinner ZIF-8 membranes were prepared with high CO_2_ permeances, ~2.4 × 10^−5^ mol/m^2^ s Pa with CO_2_/CH_4_ separation selectivities of ~4 to 7, for natural gas sweetening applications [13]. Similarly, Bio-MOF membranes were fabricated via a secondary seeded growth mechanism and showed promising CO_2_ separation performance. These innovative material membranes have led to significant improvements in the efficiency of gas separation processes.

Professor Carreon’s study examined steam methane reforming (SMR) using a palladium-based membrane reactor in collaboration with other research groups. The reactor produced ultra-high-purity hydrogen with up to 40% methane conversion under moderate conditions without sweep gas, showing high hydrogen selectivity [14]. This work demonstrated excellent performance in separating hydrogen from gas mixtures, making them suitable for hydrogen production and purification applications.

Another groundbreaking research contribution by Professor Carreon is the separation of Krypton (Kr) from Xenon (Xe) for the treatment of spent nuclear fuel. The main objective of Carreon’s work is the development of continuous crystalline microporous molecular sieve membranes to separate Kr/Xe. Fundamental understanding of the Kr/Xe separation mechanism using molecular simulations helped to develop several porous materials for this application [15]. SAPO-34 [16], ZIF-8 [17], and AlPO-18 [18] membranes have shown high Kr permeance and separation selectivity. The key factors affecting the separation selectivity and permeance of these membranes were identified and decoupled. The presence of rigid micropores with sizes lying between the Kr and Xe atomic sizes, lower Xe/Kr uptakes (adsorption selectivity), and lower concentrations of nonselective pores led to the highest observed Kr/Xe separation selectivity. This work represents one of the first known examples of microporous crystalline membranes with molecular sieving properties to separate Kr/Xe from spent nuclear fuel.

## 3. Concluding Remarks

Moises A. Carreon’s story is not just one of personal success, but of a relentless drive to push the boundaries of what is possible in material science. Moises remained actively involved in research until the last days of his life. This shows his love for research and helping his students and the scientific community. Prestigious awards received by Professor Carreon are a testament to the widespread recognition of his work and the high regard in which he is held by his peers. This Moises A. Carreon Commemorative Special Issue stands as a worthy tribute to an innovator whose work in membrane and material sciences has made a lasting impact. As we reflect on his achievements, we are reminded of the profound impact that one individual can have on the world and are inspired to continue the journey of material discovery that Professor Moises A. Carreon so passionately championed.
